# 1108. Evaluation of Vancomycin Dosing in Adolescents

**DOI:** 10.1093/ofid/ofab466.1302

**Published:** 2021-12-04

**Authors:** Ashley I Weaver, Genene A Wilson, Emily Belarski, Allison Nelson, Madan Kumar, Palak Bhagat

**Affiliations:** 1 University of Chicago Medicine/, Indianapolis, Indiana; 2 Dell Children’s Medical Center, Austin, Texas; 3 University of Chicago Medicine, Chicago, Illinois; 4 University of Chicago Medicine Comer Children’s Hospital, Geneva, IL; 5 University of Chicago, Chicago, IL

## Abstract

**Background:**

Pediatric vancomycin dosing varies based on age and renal function. Recent literature suggests previously recommended doses of 45-60 mg/kg/day may be insufficient to achieve an AUC:MIC ratio of 400-600 mg-hr/L and higher doses of at least 60 mg/kg/day may be required. However, data to guide dosing in adolescents is limited.

**Methods:**

A single-center, retrospective chart review of patients aged 12 to 18 years who received vancomycin and had therapeutic drug monitoring (TDM) performed between July 2017 to June 2020 were included. The primary endpoint was the median total daily dose (TDD) of vancomycin required to achieve therapeutic serum concentrations. Secondary endpoints were to characterize how factors such as age, weight, trough versus AUC monitoring, malignancy, and trauma may influence dosing. The safety endpoint was the development of acute kidney injury (AKI).

**Results:**

130 vancomycin courses in 86 patients were included. Baseline characteristics are presented in Table 1. Of the 130 vancomycin courses, 50 courses (38%) achieved therapeutic serum concentrations at a median TDD of 49.8 mg/kg/day (IQR 42 – 59.4). This was not statistically different from the sub- or supra-therapeutic groups (p=0.22). Based on age, the median TDD for 12-14 year olds was higher at 60 mg/kg/day (IQR 45-78.8; n=14) than for 15-16 and 17-18 year olds [45.3 mg/kg/day (IQR 41.1-51; n=15), 48 mg/kg/day (IQR 42-52; n=21), respectively]. Obese patients needed a median TDD of 43.5 mg/kg/day vs at least 51 mg/kg/day in healthy and overweight patients. Finally, AUC guided dosing resulted in a slightly lower overall median TDD vs trough guided dosing (45.8 mg/kg/day vs 50.5 mg/kg/day). Additional dose requirements based on age, weight, TDM and other characteristics are presented in Table 2. Of the 15 patients who developed AKI per pRIFILE criteria, 2 were classified as injury and 3 as failure.

Table 2. Total Daily Dose Course Analysis

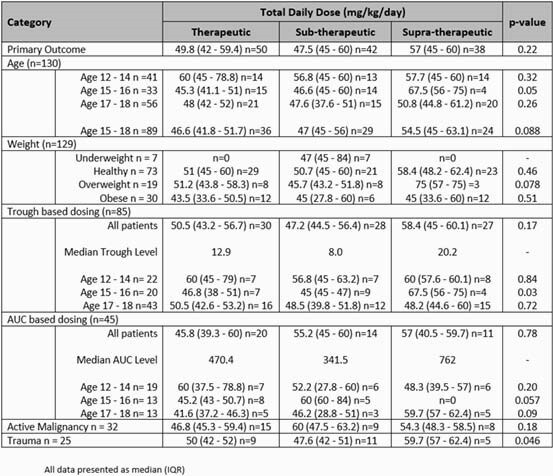

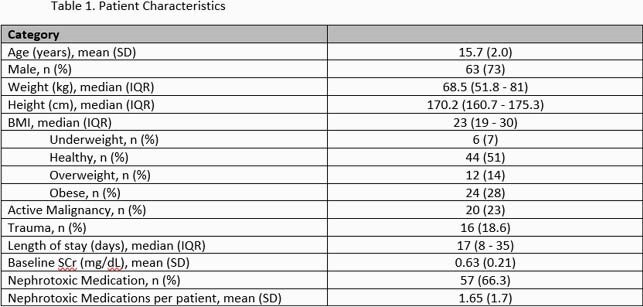

**Conclusion:**

To achieve therapeutic levels, adolescents 12 to14 years old need higher empiric doses of 60 mg/kg/day compared to 45 mg/kg/day in 15 to 18 year olds. Obese patients, however, may require lower TDD than underweight, healthy, and overweight patients. Patients that receive AUC versus trough monitoring may also require lower TDD to achieve therapeutic concentrations. More data is needed to further evaluate our findings.

**Disclosures:**

**All Authors**: No reported disclosures

